# Supplementary material for: Sub-Lexical Processing of Chinese–English Bilinguals: An ERP Analysis
Source: Brain Sci. 2024 Sep 16;14(9):923. doi: 10.3390/brainsci14090923 (PMC11430797; doi:10.3390/brainsci14090923)
Supplement: Supplementary file 1 [file brainsci-14-00923-s001.zip › brainsci-3170816-supplementary.pdf]

Table S1 Stimuli for the Semantic Relatedness Task

Prime	Target (+S, +F)	Translation (+S, +F)	Target (-S, +F)	Translation (-S, +F)	Target (+S, -F)	Translation (+S, -F)	Target (-S, -F)	Translation (-S, -F)	Semantic Radical
water	ocean	海洋	desert	沙漠	beverage	饮料	soccer	足球	氵 water/liquid related
water	swim	游泳	activate	激活	milk	牛奶	prince	王子	氵 water/liquid related
water	river	河	law	法	rain	雨	foot	脚	氵 water/liquid related
water	soup	汤	hole	洞	snow	雪	ghost	鬼	氵 water/liquid related
water	liquor	酒	actor	演员	coffee	咖啡	history	历史	氵 water/liquid related
jewelry	pearl	珍珠	rose	玫瑰	diamond	钻石	bread	面包	王 jade/jewelry/treasure related
jewelry	glass	玻璃	ball	球	crystal	水晶	television	电视	王 jade/jewelry/treasure related
jewelry	amber	琥珀	play	玩	gold	金	classroom	教室	王 jade/jewelry/treasure related
mouth	drink	喝	leaf	叶子	tooth	牙	war	战争	口 mouth related
mouth	breath	呼吸	ton	吨	laugh	笑	airplane	飞机	口 mouth related
mouth	eat	吃	listen	听	face	脸	salt	盐	口 mouth related
tree	cherry	樱桃	camera	相机	seed	种子	hospital	医院	木 tree/wood related
tree	lemon	柠檬	cup	杯子	potato	土豆	difficulty	困难	木 tree/wood related
tree	forest	森林	village	村子	cucumber	黄瓜	health	健康	木 tree/wood related
tree	peach	桃子	bridge	桥	grass	草	dinner	晚饭	木 tree/wood related
tree	root	根	gun	枪	flower	花	phone	电话	木 tree/wood related
tree	coconut	椰子	template	模板	banana	香蕉	egg	鸡蛋	木 tree/wood related
tree	orange	橙子	pillow	枕头	strawberry	草莓	fridge	冰箱	木 tree/wood related
tree	olive	橄榄	mechanical	机械	peanut	花生	candle	蜡烛	木 tree/wood related
tree	walnut	核桃	stairs	楼梯	bamboo	竹子	marriage	婚姻	木 tree/wood related
tree	maple	枫树	brown	棕	pumpkin	南瓜	shirt	衬衫	木 tree/wood related
person	idol	偶像	value	价值	friend	朋友	moon	月亮	亻 people related
person	partner	伙伴	underestimate	低估	teacher	老师	dragon	龙	亻 people related
person	buddha	佛	fake	假	doctor	医生	north	北	亻 people related
language	comment	评论	design	设计	song	歌曲	window	窗户	讠 language related
language	discussion	讨论	honesty	诚实	communicate	交流	truck	卡车	讠 language related
language	poem	诗	trick	诡计	report	报告	floor	地板	讠 language related

Prime	Target (+S, +F)	Translation (+S, +F)	Target (-S, +F)	Translation (-S, +F)	Target (+S, -F)	Translation (+S, -F)	Target (-S, -F)	Translation (-S, -F)	Semantic Radical
thread	fiber	纤维	continue	继续	craft	手工	freedom	自由	纟 sewing/thread related
thread	sew	缝	green	绿	needle	针	car	汽车	纟 sewing/thread related
thread	weave	编织	red	红	cloth	布	butterfly	蝴蝶	纟 sewing/thread related
thread	rope	绳子	give	给	cotton	棉花	juice	果汁	纟 sewing/thread related
thread	knot	结	pure	纯	wool	羊毛	table	桌子	纟 sewing/thread related
thread	twine	缠绕	New York	纽约	nylon	尼龙	candy	糖果	纟 sewing/thread related
plant	tea	茶	bitter	苦	watermelon	西瓜	speed	速度	艹 plant related
plant	vegetable	蔬菜	ballet	芭蕾	pear	梨	piano	钢琴	艹 plant related
plant	grape	葡萄	blue	蓝	corn	玉米	fox	狐狸	艹 plant related
plant	mint	薄荷	festival	节日	ginger	姜	scarf	围巾	艹 plant related
plant	mushroom	蘑菇	art	艺术	pea/bean	豆	tennis	网球	艹 plant related
emotion	pleasant	愉快	lazy	懒	excited	激动	young	年轻	忄 emotion/feeling related
emotion	hate	恨	busy	忙	angry	生气	black	黑	忄 emotion/feeling related
emotion	fear	怕	fast	快	love	爱	short	短	忄 emotion/feeling related
emotion	repent	忏悔	generous	慷慨	satisfied	满足	far	远	忄 emotion/feeling related
horse	donkey	驴	deceiver	骗子	deer	鹿	computer	电脑	马 horse related
disease	cancer	癌症	thin	瘦	flu	流感	lock	锁	疒 disease related
metal	silver	银	wrong	错	sword	剑	map	地图	钅 metal related
metal	iron	铁	town	镇子	knife	刀	shirt	衬衫	钅 metal related
animal	dog	狗	guess	猜	cow	牛	boat	船	犭 animal related
animal	cat	猫	independence	独立	horse	马	train	火车	犭 animal related

Table S2 Orthographic Similarity (Levenshtein Distance) Scores of Prime-Target Pairs in the Semantic Relatedness Task.

+S+F	-S+F	+S-F	-S-F
5	4	6	4
5	6	5	6
3	4	4	5
5	4	5	5
5	3	5	5
5	6	7	6
7	6	7	8
5	5	6	8
5	5	2	5
4	4	3	8
5	5	5	4
5	5	3	8
5	4	5	10
4	6	7	6
5	4	4	5
4	4	5	4
7	6	6	4

+S+F	-S+F	+S-F	-S-F
4	6	7	4
4	10	6	5
6	5	6	6
4	4	7	5
5	6	5	4
5	10	6	4
6	6	5	5
8	7	6	7
9	7	8	7
8	8	8	8
5	8	5	5
5	4	6	5
5	3	6	7
5	5	6	5
6	4	6	6
5	8	6	5
4	6	9	5
8	4	3	2
4	4	4	5
3	8	6	4
8	3	3	5
7	7	6	6
6	7	7	7
7	6	6	6
7	7	8	7
4	7	5	6
7	7	7	7
6	5	5	3
5	5	5	5
6	6	6	5
5	11	6	6

Table S3. Concreteness Scores of the Target Stimuli in the Semantic Relatedness Task.

+S+F	-S+F	+S-F	-S-F
4.86	4.86	4.6	4.76
4.43	2.97	4.92	4.44
4.89	2.57	4.97	4.9
4.72	4.81	4.85	3.19
4.73	4.57	4.81	2.96
4.87	4.9	4.89	4.92
4.82	5	4.58	4.83
4.35	3.24	4.81	4.76
4.76	5	4.89	3.63
4.36	4.17	4.21	4.96
4.44	3.47	4.87	4.89
4.62	5	4.71	4.64
5	5	4.85	1.9
4.76	4.89	4.83	2.28
4.9	4.97	4.93	4.5
4.34	4.83	5	4.86
4.79	4.19	5	4.97
4.66	5	5	4.92
4.9	3.17	4.89	4.86
4.97	5	4.86	2.51
4.46	4.48	4.9	4.94
3.63	1.62	3.07	4.9
3.53	2.04	4.52	4.39
NA	1.97	4.69	4.14
3.29	3.37	4.46	4.86
3.07	1.58	3.43	4.84

+S+F	-S+F	+S-F	-S-F
4.03	3.36	3.92	4.8
4.5	2.36	3.48	2.34
3.93	4.07	4.93	4.89
4.2	4.24	4.9	4.93
4.93	2.83	4.97	4.89
4.87	1.87	4.86	4.9
4.03	1.41	4.7	4.83
4.69	2.93	4.89	3.62
4.89	4.04	4.93	4.9
5	3.76	4.96	4.97
4.54	4.33	4.92	4.97
4.83	4.17	5	4.43
1.55	2.67	2.32	3.16
1.97	2.41	2.53	3.76
2.57	3.32	2.07	3.61
1.96	2.25	2.07	2.71
5	2.66	4.86	4.93
3.86	3.83	3.65	4.65
4.52	1.83	4.93	4.93
4.59	4.64	4.9	4.94
4.85	2.28	4.96	4.93
4.86	1.87	5	4.79
